# Variability of Emaravirus Species Associated with Sterility Mosaic Disease of Pigeonpea in India Provides Evidence of Segment Reassortment

**DOI:** 10.3390/v9070183

**Published:** 2017-07-11

**Authors:** Basavaprabhu L. Patil, Meenakshi Dangwal, Ritesh Mishra

**Affiliations:** ICAR-National Research Centre on Plant Biotechnology, IARI, Pusa Campus, New Delhi 110012, India; meenakshi.dangwal@gmail.com (M.D.); biotech2ritesh@gmail.com (R.M.)

**Keywords:** pigeonpea sterility mosaic virus, emaravirus, variability, reassortment

## Abstract

Sterility mosaic disease (SMD) of pigeonpea is a serious constraint for cultivation of pigeonpea in India and other South Asian countries. SMD of pigeonpea is associated with two distinct emaraviruses, Pigeonpea sterility mosaic virus 1 (PPSMV-1) and Pigeonpea sterility mosaic virus 2 (PPSMV-2), with genomes consisting of five and six negative-sense RNA segments, respectively. The recently published genome sequences of both PPSMV-1 and PPSMV-2 are from a single location, Patancheru from the state of Telangana in India. However, here we present the first report of sequence variability among 23 isolates of PPSMV-1 and PPSMV-2, collected from ten locations representing six states of India. Both PPSMV-1 and PPSMV-2 are shown to be present across India and to exhibit considerable sequence variability. Variability of RNA3 sequences was higher than the RNA4 sequences for both PPSMV-1 and PPSMV-2. Additionally, the sixth RNA segment (RNA6), previously reported to be associated with only PPSMV-2, is also associated with isolates of PPSMV-1. Multiplex reverse transcription PCR (RT-PCR) analyses show that PPSMV-1 and PPSMV-2 frequently occur as mixed infections. Further sequence analyses indicated the presence of reassortment of RNA4 between isolates of PPSMV-1 and PPSMV-2.

## 1. Introduction

Two emaravirus species, *Pigeonpea sterility mosaic emaravirus 1* (PPSMV-1) and *Pigeonpea sterility mosaic emaravirus 2* (PPSMV-2), are associated with sterility mosaic disease (SMD) of pigeonpea (*Cajanus cajan* (L.) Millsp) [1,2,3]. Sterility mosaic disease is one of the major constraints for cultivation of pigeonpea in India and is endemic to India, Nepal, Bangladesh and Myanmar [4,5]. The characteristic symptoms of SMD are complete-to-partial cessation of flowering (sterility), excessive vegetative growth, stunting, chlorotic rings or mosaic symptoms on the leaves and a reduction in leaf size [6]. However, the nature and severity of symptoms largely depend on pigeonpea genotype and plant age at the time of virus infection [6]. The viruses involved in SMD are transmitted by the eriophyid mite *Aceria cajani* Channabassavanna (Acari:Arthropoda) and are not known to be seed-transmitted [7,8].

Though SMD of pigeonpea was first reported in 1931 from the state of Bihar in India [4], the genome sequences of the two emaraviruses associated with this disease have only recently been published [1,2]. Based on the genome organization and sequence information of PPSMV-1 and PPSMV-2, they are taxonomically included in the genus *Emaravirus* in the recently created family *Fimoviridae* within the order *Bunyavirales* [9,10,11]. *Emaravirus* comprises economically important plant viruses with negative-sense segmented RNA genomes, consisting of 4–8 RNA segments depending on the species [11,12]. *European mountain ash ringspot-associated emaravirus* (EMARaV) is the type species of the genus *Emaravirus* with *Fig mosaic emaravirus* (FMV), *Raspberry leaf blotch emaravirus* (RLBV), *Rose rosette emaravirus* (RRV), *Redbud yellow ringspot associated emaravirus* (RYRSaV), *Actinidia chlorotic ringspot-associated emaravirus* (AcCRaV), and *High plains wheat mosaic emaravirus* (HPWMoV) as other definitive members, which have been recently ratified and renamed [9,12,13,14,15,16,17].

The first sequence of PPSMV published, later renamed PPSMV-1, was reported to contain five genomic RNA segments of 7022 nt, 2223 nt, 1442 nt, 1563 nt and 1689 nt coding for an RNA-dependent RNA polymerase (RdRp), a glycoprotein (GP), a nucleocapsid protein (NP), a movement protein (MP) and p5, respectively [1,2]. Subsequently, another emaravirus species, PPSMV-2, was reported to be associated with SMD of pigeonpea [2]. The first four RNA segments of PPSMV-2 share higher sequence similarity with FMV than with PPSMV-1 [1,2]. In contrast to the presence of five RNA segments in PPSMV-1, PPSMV-2 was associated with six RNA segments [1,2]. The additional RNA segment of 1194 nt, referred to as RNA6, encodes a protein of unknown function [2].

The available sequences of PPSMV-1 and PPSMV-2 are from Patancheru (Greater Hyderabad) in the state of Telangana, India. However, SMD in India was also reported from Andhra Pradesh, Bihar, Gujarat, Karnataka, Maharashtra, Tamil Nadu, Telangana and Uttar Pradesh states (Supplementary Figure S2) [3,8]. RNA viruses are highly heterogeneous in nature, because of their huge population sizes, short multiplication time, and high rates of mutation as a result of error-prone replication by the RdRp [18,19,20]. Further, recombination and reassortment among the segments of viral RNA genomes significantly contribute to the sequence variability [21,22]. Some plant RNA viruses evolve rapidly, while others evolve slowly resulting in either genetically diverse or stable virus populations [23,24,25]. A thorough understanding of the genetic variability of emaraviruses involved in SMD of pigeonpea may help in the development of reliable and robust diagnostic tools and improved disease management strategies [13,26,27,28,29,30,31]. The sequence variability among the 23 isolates of PPSMV-1 and PPSMV-2, collected from ten geographic locations across India, was assessed by producing complete/partial sequences of selected RNA segments (Supplementary Table S1). The samples were also screened for the presence of RNA6. A diagnostic multiplex-RT-PCR protocol was also developed to detect and distinguish PPSMV-1 and PPSMV-2.

## 2. Materials and Methods

### 2.1. Collection of SMD-Affected Pigeonpea Leaf Samples

Leaf samples from 23 pigeonpea plants with typical SMD symptoms were collected from 10 locations in India, during 2013–2015. The locations are Bidar, Mahagaon, Kalaburagi (erstwhile Gulbarga), Raichur and Bengaluru (erstwhile Bangalore) in the state of Karnataka, Coimbatore in Tamil Nadu state, Pune in the state of Maharashtra, Dholi in the state of Bihar, Gorakhpur in Uttar Pradesh state and from New Delhi (Supplementary Table S1, Supplementary Figures S1 and S2). Symptomatic samples along with healthy controls were brought to New Delhi in cold packs, snap frozen in liquid nitrogen and stored in a −80 °C freezer until further use. The presence of PPSMV-1 and PPSMV-2 in the leaf samples was confirmed by using emaravirus-specific degenerate PCR primers, which targeted conserved motifs of the RdRp [32].

### 2.2. RNA Extraction, RT-PCR and Cloning of PPSMV-1 and PPSMV-2 Segments

About 100 mg of symptomatic and healthy pigeonpea leaf tissues were used for total RNA extraction, by crushing them under liquid nitrogen. Total RNA was extracted from the leaf powder using a Spectrum Plant Total RNA Kit (Sigma-Aldrich, St. Louis, MO, USA) following the manufacturer’s instructions. Total RNA (2 μg) was reverse-transcribed using a MultiScribe Reverse Transcriptase, RT Random Primers and other components from the High-Capacity cDNA Reverse Transcription kit (Applied Biosystems, Foster City, CA, USA). The reactions were carried out in a total reaction volume of 20 μL, with a cDNA synthesis cycle of 25 °C for 10 min, 37 °C for 120 min and 85 °C for 5 min, set up in a Bio-Rad S1000 Thermal cycler (Bio-Rad Laboratories, Hercules, CA, USA). The resulting cDNA was diluted 1:10 and 1 μL of the diluted cDNA was used for PCR amplification of various segments of PPSMV-1 and PPSMV-2 isolates, using specific primer pairs designed in this study (Supplementary Table S2).

The RT-PCR amplified products were resolved in 1% TAE (Tris-acetate-EDTA) agarose gels stained with ethidium bromide and visualized under ultraviolet (UV)-light. DNA fragments were eluted from the agarose gel using a QIAquick Gel Extraction Kit (Qiagen, Hilden, Germany) and were then ligated in the pGEM-T Easy vector (Promega, Madison, WI, USA) according to the manufacturer’s instructions and subsequently transformed in Escherichia coli DH5α. The recombinant plasmids containing target sequences were confirmed by colony PCR and restriction analysis, and subjected to Sanger sequencing. All pigeonpea samples were screened for the presence of RNA6 segment by PCR using RNA6-specific primer pair SMD6-F and SMD6-R (Supplementary Table S2). The RNA6 segments of selected isolates of PPSMV-1 and PPSMV-2 were cloned in pGEM-T Easy vector and sequenced.

### 2.3. Sequence Analysis of RNA Fragments of PPSMV-1 and PPSMV-2 Isolates

The raw nucleotide sequences were edited to remove vector sequences and contigs were manually assembled by identifying overlapping nucleotide sequences. Later, the nucleotide homology searches were done with the BLASTN Sequence Analysis of NCBI (http://blast.ncbi.nlm.nih.gov/Blast.cgi). The PPSMV sequences obtained by Sanger sequencing were submitted to GenBank under accession numbers KT862838-KT862842, KX363886-KX363948, KX452690-KX452693, KX458110, KX458111 and KX608992 (Supplementary Table S1). Nucleotide and deduced amino acid sequences of equal lengths of the segments of PPSMV-1 and PPSMV-2 isolates from this study, along with published sequences of PPSMV-1, PPSMV-2 and selected emaraviruses from NCBI GenBank were aligned using MUSCLE [33]. Phylogenetic trees were constructed by the maximum-likelihood [34], applying the JTT matrix and pairwise gap deletion options implemented in MEGA6 [35]. Branches with bootstrap values less than 70% were collapsed. Nucleotide and amino acid sequence identities were calculated using Bioedit [36] and similarity identity matrixes were drawn for both nucleotide and amino acid sequences of NP and MP of PPSMV-1 and PPSMV-2 isolates (Supplementary Tables S3 and S4).

The recombination detection program package RDP4 [37] was used to identify parent-like emaravirus sequences. Recombination breakpoints were identified using the inbuilt methods of the RDP4 package, such as RDP, GENECONV, BOOTSCAN, MAXIMUM CHI SQUARE, CHIMAERA, SISTER SCAN and 3SEQ, [37]. All the partial/complete sequences of the genomic RNA segments of PPSMV-1 and PPSMV-2 isolates, along with selected emaravirus sequences (FMV, EMARaV, RYRV and RLBV) were subjected to RDP analysis. The analyses were done with default settings and with a Bonferroni correction *p*-value cut-off of 0.05 (Table 1). Recombination events were considered significant only if the *p*-values were less than 1 × 10^−6^ in at least three of the seven methods of RDP4 package.

### 2.4. Diagnostic Multiplex-RT-PCR for Detection of PPSMV-1 and PPSMV-2 Isolates

To differentiate the two species, a common degenerate forward primer was designed for a conserved sequence of RNA1 of both PPSMV-1 and PPSMV-2 isolates and two differentiating degenerate reverse primers were designed targeting the variable sequence of RNA1. The primer combination dRNA1-1&2F (3931–3954 nt): CATTGTATAACACTAAATGAAAAN, dRNA1-1R (4653–4632 nt): CTAACATTCGATTCATTAGCTN and dRNA1-2R (5135–5113 nt): TGTCTAGATGTTAAAAATGATTN targeting the RNA1 segment of PPSMV-1 and PPSMV-2 producing two distinct-sized amplicons was used for all the subsequent multiplex-RT-PCR studies to detect and distinguish PPSMV-1 and PPSMV-2 in SMD-affected pigeonpea samples. The diagnostic PCR involved an initial denaturation at 95 °C for 5 min, followed by 40 cycles at 95 °C for 60 s, 54 °C for 60 s and 72 °C for 60 s and a final extension of 72 °C for 5 min.

## 3. Results

### 3.1. Analysis of NP and MP Sequence Identity between PPSMV-1 and PPSMV-2 Isolates

Estimation of percentage sequence identity among the 17 NP and 23 MP sequences of both the pigeonpea-infecting emaravirus species, along with published PPSMV sequences from Patancheru [1,2] revealed significant sequence variability (Supplementary Tables S3 and S4). The identity among the NP sequences of PPSMV-1 isolates was in the range of 86.5%–99.1% at nucleotide level and 91.5%–100.0% at amino acid level (Supplementary Table S3). Whereas in the case of PPSMV-2 isolates, the sequence identities were 86.7%–99.8% at nucleotide level and 90.7%–99.6% at amino acid level (Supplementary Table S4). Similarly, sequence identities for MP sequences of PPSMV-1 isolates was in the range of 90.8%–100.0% at nucleotide level and 94.7%–100.0% at amino acid level (Supplementary Table S4). While for PPSMV-2 isolates, the sequence identity varied from 94.1%–99.0% at nucleotide level, and 96.3%–99.7% at amino acid level (Supplementary Table S4).

### 3.2. RNA6 is Also Associated with Isolates of PPSMV-1

**Figure 1:**
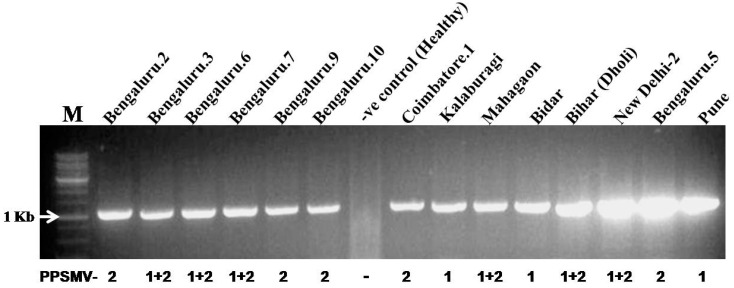
Reverse transcription PCR (RT-PCR) amplification for the detection of genomic segment RNA6 from sterility mosaic disease (SMD)-affected pigeonpea samples. The Pigeonpea sterility mosaic virus (PPSMV) species infecting each sample is indicated at the bottom of the gel, as 1 for PPSMV-1, 2 for PPSMV-2 and 1 + 2 for mixed infection of PPSMV-1 and PPSMV-2. The lane marked as “M” on the left of the gel is 1 Kb DNA size marker.

Screening of the 23 SMD-affected pigeonpea samples by RT-PCR with specific primer pairs revealed the presence of RNA6 in all the samples (Supplementary Table S2, Figure 1). The association of RNA6 with both pigeonpea-infecting emaravirus species was confirmed by cloning and sequencing of RNA6 from six selected samples, of which three were infected with PPSMV-2, two were infected with PPSMV-1 and one was infected with both viruses (Supplementary Table S1). Sequence comparison of RNA6 from PPSMV-1 and 2 revealed greater than 93% sequence identity of both the nucleotide and amino acid sequences. This suggests that a conserved function in virus biology is being performed by the RNA6 encoded protein for both PPSMV-1 and 2.

### 3.3. Recombination Analysis for Sequences of PPSMV-1 and PPSMV-2 Isolates

RDP4 analysis of PPSMV-1 and PPSMV-2 sequences indicated presence of recombinations in the genomic segments RNA1, RNA2, RNA4 and RNA5 (Table 1). However, no recombination breakpoints were detected in RNA3 and RNA6 segments of PPSMV-1 and PPSMV-2. However, the central region of RNA1 (4600–5740 nt) of PPSMV-1 Bihar isolate had a potential inter-species recombination at 5403–5495 nt, for which the major parent was Mahagaon isolate of PPSMV-1 and the minor parent was Patancheru isolate of PPSMV-2 (Table 1, Supplementary Table S1). Although the RNA2 and RNA4 segments of PPSMV-1 isolates from Pune and Mahagaon respectively showed one recombination each, with PPSMV-1 isolates as major and minor parents, the recombination was significant for only two methods (Table 1). Similarly, an inter-species recombination detected for RNA5 segment of PPSMV-1 Bihar isolate, with mixed infection, was significant for only two methods. However, for RNA5 segment of the PPSMV-2 Patancheru isolate, both the major and minor parents involved in recombination were PPSMV-1 isolates (Table 1).

### 3.4. Phylogenetic Analysis of PPSMV-1 and PPSMV-2 Sequences

**Figure 2:**
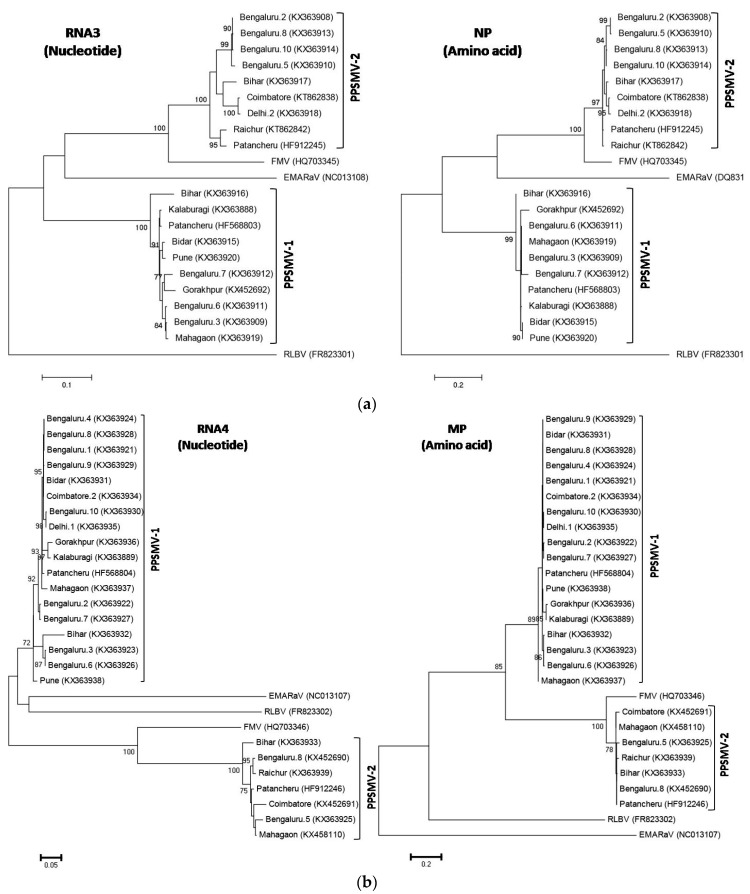
(**a**,**b**). Phylogenetic analysis of the nucleotide sequences of RNA3 and RNA4 and the amino acid sequences of the nucleocapsid protein (NP) encoded by RNA3 and the putative movement protein (MP) encoded by RNA4 of PPSMV-1 and PPSMV-2 along with the corresponding sequences of selected emaraviruses, such as *European mountain ash ringspot-associated emaravirus* (EMARaV), *Fig mosaic emaravirus* (FMV), and *Raspberry leaf blotch emaravirus* (RLBV). The GenBank accession number for each sequence is given in parentheses. Sequence alignments were done using ‘‘MUSCLE’’ and the phylogenetic trees were drawn by the maximum-likelihood, applying the JTT matrix and pairwise gap deletion options implemented in MEGA6, using 1000 bootstrap replicates. Bootstrap values greater than 70% are given at each node. The scale bar represents 0.2 substitutions per amino acid position for NP and MP sequences and 0.1 and 0.05 substitutions per nucleotide position for RNA3 and RNA4 sequences, respectively.

Out of the 17 sequences obtained for the RNA3 segment, nine were PPSMV-1 and eight were PPSMV-2, and except for two partial sequences, we could translate all into amino acid sequences (Supplementary Table S3). When the nucleotide and amino acid sequences of RNA3 or NP were subjected to phylogenetic analysis, along with selected emaravirus sequences, the isolates of PPSMV-1 and PPSMV-2 formed two separate and distinct clusters (Figure 2a). The RNA3 sequence of FMV also clustered with PPSMV-2 isolates. All the nucleotide and amino acid sequences of PPSMV-2 isolates from Bengaluru clustered together into a distinct subcluster and the PPSMV-2 isolates from Bihar, Coimbatore and Delhi clustered together. Whereas the RNA3 of Bihar isolate of PPSMV-1 distinctly separated out from all the other sub clusters of PPSMV-1 (Figure 2a). Similarly, when the RNA4 sequences of 17 PPSMV-1 isolates and six PPSMV-2 isolates were subjected to phylogenetic analysis, PPSMV-1 and PPSMV-2 formed distinct clusters (Figure 2b). The RNA4 nucleotide sequences of PPSMV-1 from Pune and PPSMV-2 from Bihar were distinctly basal to each cluster (Figure 2b). However, this was not the case for corresponding amino acid sequences of their MPs.

Phylogenetic analysis of seven RNA6 nucleotide sequences of PPSMV-1 and PPSMV-2, respectively, showed a region-specific clustering, irrespective of their association with either PPSMV-1 or PPSMV-2 isolates (Supplementary Figure S3). The RNA6 sequence from Bihar isolate was phylogenetically the most diverse, showing highest sequence identity (93.0%) with Patancheru isolate of PPSMV-2 (Supplementary Figure S3). However, it was unclear whether the RNA6 sequence from Bihar belongs to PPSMV-1 or PPSMV-2, since the plant was infected with both viruses.

### 3.5. Diagnostic Multiplex-RT-PCR and Distribution of PPSMV-1 and PPSMV-2 

**Figure 3:**
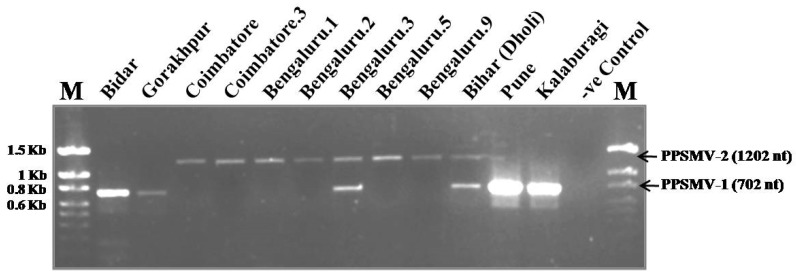
Multiplex-RT-PCR for detection and differentiation of PPSMV-1 and PPSMV-2 isolates from SMD-affected pigeonpea samples. The lanes marked as “M” on the left and right extremes of the gel are the 100 bp DNA size marker (G Biosciences, St. Louis, MO, USA) for which the lengths of each bands are indicated. The two arrows point to the PPSMV-1 and PPSMV-2 specific amplicons and their sizes are also given.

The primer combination targeting RNA1 segment resulted in amplicons of two distinct sizes; 702 nt for PPSMV-1 and 1202 nt for PPSMV-2 isolates (Figure 3). The multiplex-RT-PCR analysis of 23 SMD-affected pigeonpea leaf samples, collected from ten locations covering six states of India, showed that PPSMV-1 and PPSMV-2 are present in both northern and southern parts of India and also that they occur as mixed infections (Supplementary Figures S1 and S2). Previously published reports about the occurrence of both PPSMV-1 and PPSMV-2 isolates as single and mixed infection in Patancheru, in the state of Telangana are also indicated (Supplementary Figure S2) [1,2].

### 3.6. Reassortment of RNA4 Segment from PPSMV-1 to PPSMV-2 Isolates

Analysis of the virus sequences of five SMD-affected samples from Bengaluru (Bengaluru.1, Bengaluru.2, Bengaluru.4, Bengaluru.9 and Bengaluru.10) and one from Coimbatore revealed the exchange of RNA4 from PPSMV-1 to PPSMV-2. The RNA1 and RNA3 segments of these isolates show highest levels of sequence similarities to the homologous components of PPSMV-2. Whereas the RNA4 shows highest levels of sequence similarities to the homologous components of PPSMV-1 and also segregate with the PPSMV-1 isolates when subjected to phylogenetic analysis (Supplementary Table S1, Figure 2b). Unfortunately, the sequences of the RNA2 and RNA5 were not obtained for these isolates. The diagnostic-multiplex-RT-PCR for the RNA1 nevertheless ruled out the possibility of mixed infections in these six samples (Figure 3). Additionally, the presence of PPSMV-2 specific RNA1 was confirmed by using specific primer pairs (Supplementary Table S2) [2]. Cloning and sequencing of the parts of RNA1 from two samples (Bengaluru.1 and Coimbatore.2) with reassortments confirmed that they were PPSMV-2 isolates which acquired RNA4 from PPSMV-1 (Supplementary Table S1).

## 4. Discussion

The study described here has, for the first time, analysed the diversity of emaraviruses associated with SMD of pigeonpea in India. This study shows that PPSMV-1 and PPSMV-2 are widespread across India and also occur as mixed infections. Analysis of sequence identity among the isolates of PPSMV-1 and PPSMV-2 indicated the presence of significant sequence variability. In particular, the sequence of PPSMV-1 isolate from Bihar (Dholi) was the most divergent of all the PPSMV-1 sequences analysed in this study. The diverse nature of the Bihar isolate was also reflected in the phylogenetic analysis of their RNA segments. The range of sequence identity for nucleocapsid protein was similar for both PPSMV-1 and PPSMV-2 isolates. However, for MP the PPSMV-1 isolates showed a wider range of sequence identities than PPSMV-2 isolates. The RNA3 sequences were more divergent than the RNA4 sequences for both PPSMV-1 and PPSMV-2. The relative differences in the extent of their sequence variability could be because of the differences in thermodynamic stability of viral RNA and differing selection pressures [19,31]. The diversity of the emaraviruses FMV [31], RLBV [26], WMoV [29] and RYRV [13] has been investigated. For RLBV isolates from Finland a nucleocapsid sequence identity of 92.0%–100.0% and 89.3%–100.0% for nucleotide and amino acid sequences, respectively, has been determined [26]. The range of sequence identities for NP of PPSMV-1 and PPSMV-2 were also similar to the reports made for NP of RLBV [26].

*Emaravirus* is one of the most recently established plant virus genera and is taxonomically placed in the newly created family *Fimoviridae*, in the order *Bunyavirales* [9]. In recent years, there have been increased reports of previously unidentified emaravirus species and their genomic RNA segments. Additional genomic segments, including RNA5 and RNA6 of FMV and RRV [12,38,39] and RNA4, RNA5 and RNA6 of RLBV, have recently been reported [40,41]. Recently eight genomic RNA segments were reported for WMoV [16]. Moreover, this emaravirus is shown to be associated with two types of nucleocapsid proteins with unusual heterogeneity [16]. The study here shows that RNA6 is also associated with isolates of PPSMV-1, which is in contrast to previous reports of the association of RNA6 only with isolates of PPSMV-2 [1,2]. The diagnostic multiplex-RT-PCR analyses rule out the possibility of mixed infections in the PPSMV-1 infected pigeonpea samples positive for RNA6. Sequence analyses show that RNA6 is the most conserved genomic segment of both PPSMV-1 and PPSMV-2. Recombination break points were detected for RNA1, RNA2, RNA4 and RNA5 segments, but not for RNA3 and RNA6. This is also a first report of possible inter-species recombination between PPSMV-1 and PPSMV-2 segments. 

The NP amino acid sequences of PPSMV-2 isolates and the MP amino acid sequences of PPSMV-1 isolates from Bengaluru were phylogenetically closer. Interestingly, a geographical segregation was observed when the nucleotide sequences of RNA6 were subjected to phylogenetic analysis, irrespective of whether the isolates were PPSMV-1 or PPSMV-2. In contrast to RNA3 and RNA4 sequences, the RNA5 and RNA6 sequence of FMV did not cluster with the isolates of PPSMV-2, indicating that RNA5 and RNA6 of PPSMV-1 and PPSMV-2 diverged very recently from a common ancestor; alternatively, the two species may be frequently exchanging these segments. Previously, emaravirus-specific degenerate primers were developed against the conserved motifs of RdRp sequences, which also amplified partial sequence of PPSMV RNA1 [32]. However, in this study we developed and optimised a diagnostic multiplex-RT-PCR method to detect and also to differentiate the isolates of PPSMV-1 and PPSMV-2. This diagnostic RT-PCR was used to study the frequency of infection and geographic distribution of PPSMV-1 and PPSMV-2. The results indicate that PPSMV-1 is more prevalent in northern parts of Karnataka, Maharashtra and the three states of north India. In contrast, PPSMV-2 is more prevalent in Coimbatore and Bengaluru in southern India. However, a larger number of samples need to be analysed to confirm this finding. The highest frequencies of mixed PPSMV-1/PPSMV-2 infections were in Bengaluru. Such information may help in developing effective management strategies for pigeonpea SMD across India, particularly by RNA-interference technology which is sequence-specific in nature [42].

These studies showed evidence for exchange of RNA4 segment from PPSMV-1 to PPSMV-2. All the reassortments were identified in PPSMV-2, which may indicate that PPSMV-2 isolates are more versatile for reassortment and is compatible with the RNA4 segment of PPSMV-1. Similarly, such segment reassortments were reported for FMV [31]. Reassortment of viral RNA segments can provide fitness advantages to the progeny viruses, or reduce the fitness of a virus depending on the gain or loss of the beneficial alleles [21,43]. Reassortment is also reported for other segmented RNA viruses infecting both plants and animals. The plant infecting viruses for which reassortment has been reported are Brome mosaic virus (*Bromoviridae*), Tomato spotted wilt virus (*Tospoviridae*), Lettuce infectious yellows virus (*Closteroviridae*) and White clover cryptic virus 1 (*Partitiviridae*) [21,44].

Taken together, this study is the first analysis of the sequence variability of PPSMV-1 and PPSMV-2 in India, which also provides the evidence for reassortment and recombination among their genomic segments. The association of RNA-6 with the isolates of PPSMV-1 was also shown. Information on the distribution of PPSMV-1 and PPSMV-2 in India will be of significant importance in developing diagnostic tools for detection of PPSMV-1 and PPSMV-2 isolates, and also for the development of sound SMD management strategies in pigeonpea.

## Figures and Tables

**Table 1:** Summary of unique recombination breakpoints in the RNA segments of PPSMV-1 and PPSMV-2 isolates as detected by the Recombination Detection Program v.4.16 (RDP4), using seven different methods RDP, GENECONV, BOOTSCAN, MAXIMUM CHISQUARE, CHIMAERA, SISCAN, 3 SEQ. The *p*-values of all the seven methods are given; Non-Significant *p*-values are indicated as NS. Parents of the interspecies recombinations are marked in bold fonts with red colour.

PPSMV Genomic Segment		RNA 1	RNA 2	RNA 4	RNA 5	
Recombinant Isolate		PPSMV-1 Bihar	PPSMV-1 Pune	PPSMV-1 Mahagaon	PPSMV-2 Patancheru	PPSMV-1 Bihar
Recombination Breakpoint		5403–5495 nt	554–1550 nt	862–1178 nt	1228–1398 nt	453–688 nt
**Parent Isolates**	**Major**	PPSMV-1 Mahagaon	PPSMV-1 Patancheru	PPSMV-1 Kalaburagi	PPSMV-1 Kalaburagi	**PPSMV-2 Bihar**
	**Minor**	**PPSMV-2 Patancheru**	PPSMV-1 Kalaburagi	PPSMV-1 Delhi.1	PPSMV-1 Patancheru	PPSMV-1 Kalaburagi
*p*-values for 7 recombination detection methods of RDP4	**RDP**	**8.710 × 10^−07^**	NS	**4.794 × 10^−08^**	**1.432 × 10^−10^**	NS
	**GENECONV**	**3.152 × 10^−07^**	1.811 × 10^−02^	NS	**1.820 × 10^−09^**	1.877 × 10^−05^
	**BOOTSCAN**	NS	6.993 × 10^−03^	4.917 × 10^−02^	**1.134 × 10^−10^**	**1.748 × 10^−06^**
	**MAXI CHISQUARE**	NS	6.874 × 10^−04^	2.057 ×10^−02^	1.043 ×10^−03^	1.556 × 10^−04^
	**CHIMAERA**	NS	2.252 × 10^−04^	NS	9.874×10^−04^	6.743 × 10^−03^
	**SISCAN**	**2.912 × 10^−08^**	**2.404 × 10^−06^**	**3.634× 10^−06^**	**3.692 ×10^−06^**	**1.886 × 10^−10^**
	**3SEQ**	**1.454 × 10^−09^**	**5.609 × 10^−06^**	3.969 × 10^−02^	NS	2.209 × 10^−04^

## Supplementary Materials

The following are available online at www.mdpi.com/1999-4915/9/7/183/s1. Table S1: Summary of all the 23 isolates of PPSMV-1 and PPSMV-2 and their RNA segments sequenced from this study, Table S2: Primers used for RT-PCR amplification of different RNA segments of PPSMV-1 and PPSMV-2 isolates and their nucleotide coordinates, Table S3: Percent nucleotide sequence identities of nucleocapsid protein (NP) of PPSMV-1 and PPSMV-2 isolates and their corresponding amino acid sequence identities, Table S4: Percent nucleotide sequence identities of the putative movement protein (MP) of PPSMV-1 and PPSMV-2 isolates and their corresponding amino acid sequence identities, Figure S1: Symptoms of sterility mosaic disease (SMD)-affected pigeonpea from India used in this study, Figure S2: Map of India showing the ten locations of pigeonpea samples collected and distribution of PPSMV-1 and PPSMV-2, Figure S3: Phylogenetic analysis of nucleotide sequences of RNA5 and RNA6 segments of PPSMV-1 and 2 isolates.
