# Supplementary material for: Variability of Emaravirus Species Associated with Sterility Mosaic Disease of Pigeonpea in India Provides Evidence of Segment Reassortment
Source: Viruses. 2017 Jul 11;9(7):183. doi: 10.3390/v9070183 (PMC5537675; doi:10.3390/v9070183)
Supplement: Supplementary file 1 [file viruses-09-00183-s001.docx]

**Supporting Information**

**Table S1**. Summary of all the 23 isolates of PPSMV-1 and PPSMV-2 and their RNA segments sequenced from this study.

|  | **Location** | **RNA1** | **RNA3** | **RNA4** | **RNA6** |
| --- | --- | --- | --- | --- | --- |
| 1 | Bengaluru.1  **(Reassortment)** | PPSMV-2  (KX363892) | PPSMV-2  (RT-PCR) | **PPSMV-1**  (KX363921) | RT-PCR +ve |
| 2 | Bengaluru.2  **(Reassortment)** | PPSMV-2  (RT-PCR) | PPSMV-2  (KX363908) | **PPSMV-1**  (KX363922) | PPSMV-1  (KX363942) |
| 3 | Bengaluru.3  **(Mixed Infection)** | PPSMV-1  (RT-PCR) | PPSMV-1  (KX363909) | PPSMV-1  (KX363923) | RT-PCR +ve |
|  |  | PPSMV-2  (RT-PCR) | PPSMV-2  (RT-PCR) | PPSMV-2  (RT-PCR) |  |
| 4 | Bengaluru.4  **(Reassortment)** | PPSMV-2  (RT-PCR) | PPSMV-2  (RT-PCR) | **PPSMV-1**  (KX363924) | RT-PCR +ve |
| 5 | Bengaluru.5 | PPSMV-2  (RT-PCR) | PPSMV-2  (KX363910) | PPSMV-2  (KX363925) | RT-PCR +ve |
| 6 | Bengaluru.6  **(Mixed Infection)** | PPSMV-1  (KX363893) | PPSMV-1  (KX363911) | PPSMV-1  (KX363926) | RT-PCR +ve |
|  |  | PPSMV-2  (RT-PCR) | PPSMV-2  (RT-PCR) | PPSMV-2  (RT-PCR) |  |
| 7 | Bengaluru.7  **(Mixed Infection)** | PPSMV-1  (KX608992, KX363894) | PPSMV-1  (KX363912) | PPSMV-1  (KX363927) | RT-PCR +ve |
|  |  | PPSMV-2  (RT-PCR) | PPSMV-2  (RT-PCR) | PPSMV-2  (RT-PCR) |  |
| 8 | Bengaluru.8  **(Mixed Infection)** | PPSMV-1  (KX363895) | PPSMV-1  (RT-PCR) | PPSMV-1  (KX363928) | RT-PCR +ve |
|  |  | PPSMV-2  (RT-PCR) | PPSMV-2  (KX363913) | PPSMV-2  (KX452690) |  |
| 9 | Bengaluru.9  **(Reassortment)** | PPSMV-2  (RT-PCR) | PPSMV-2  (RT-PCR) | **PPSMV-1**  (KX363929) | RT-PCR +ve |
| 10 | Bengaluru.10  **(Reassortment)** | PPSMV-2  (RT-PCR) | PPSMV-2  (KX363914) | **PPSMV-1**  (KX363930) | RT-PCR +ve |
| 11 | Bidar | PPSMV-1  (RT-PCR) | PPSMV-1  (KX363915) | PPSMV-1  (KX363931) | RT-PCR +ve |
| 12 | Bihar (Dholi)  **(Mixed Infection)** | PPSMV-1  618-3798 nt  KX363896)  4019-7011nt (KX363897) | PPSMV-1  (KX363916) | PPSMV-1  (KX363932) | PPSMV-1/2  (KX363943) |
|  |  | PPSMV-2  115-566 nt  (KX363898)  5283-6016nt (KX363899) | PPSMV-2  (KX363917) | PPSMV-2  (KX363933) |  |
| 13 | Coimbatore | PPSMV-2  (KX458111) | PPSMV-2  (KT862838) | PPSMV-2  (KX4526910) | RT-PCR +ve |
| 14 | Coimbatore.1 | PPSMV-2  (KX363900) | - | - | PPSMV-2  (KX363944) |
| 15 | Coimbatore.2  **(Reassortment)** | PPSMV-2  (KX363901) | PPSMV-2  (RT-PCR) | **PPSMV-1**  (KX363934) | PPSMV-2  (KX363945) |
| 16 | Coimbatore.3 | PPSMV-2  (KX363902) | - | - | RT-PCR +ve |
| 17 | Delhi-1  **(Mixed Infection)** | PPSMV-1  (RT-PCR) | - | PPSMV-1  (KX363935) | RT-PCR +ve |
|  |  | PPSMV-2  (RT-PCR) | - | - |  |
| 18 | Delhi- 2 | PPSMV-2  (RT-PCR) | PPSMV-2  (KX363918) | - | RT-PCR +ve |
| 19 | Gorakhpur | PPSMV-1  (RT-PCR) | PPSMV-1  (KX363936) | PPSMV-1  (KX452692) | RT-PCR +ve |
| 20 | Kalaburagi  (Gulbarga) | PPSMV-1  (KX363886) | PPSMV-1  (KX363888) | PPSMV-1  (KX363889) | PPSMV-1  (KX363891) |
| 21 | Mahagaon  **(Mixed Infection)** | PPSMV-1  (KX452693) | PPSMV-1  (KX363919) | PPSMV-1  (KX363937) | RT-PCR +ve |
|  |  | PPSMV-2  (KX363903) | - | PPSMV-2  (KX458110) |  |
| 22 | Pune | PPSMV-1  (KX363904) | PPSMV-1  (KX363920) | PPSMV-1  (KX363938) | RT-PCR +ve |
| 23 | Raichur | PPSMV-2  (RT-PCR) | PPSMV-2  (KT862842) | PPSMV-2  (KX363939) | PPSMV-2  (KX363946) |

Note: For many isolates the presence of either the PPSMV-1 or PPSMV-2 specific RNA1 and RNA6 segments were confirmed by RT-PCR alone and this is indicated in parenthesis within the specific cells of this table. The samples which had mixed infections or reassortments of RNA4 (in bold) are also indicated. The NCBI accession numbers are given in parenthesis for all the sequences of the RNA segments of PPSMV-1 and PPSMV-2 isolates. Non-availability of sequence information for some of the segments is indicated by “-” and the presence of RNA6 confirmed by RT-PCR alone are indicated as RT-PCR +ve. RNA2 and RNA5 sequences are also available for PPSMV-1 isolate from Kalaburagi (Acc. No.: KX363887 and KX363890) and for both PPSMV-1 (KX363905 and KX363940) and PPSMV-2 (KX363906 and KX363941) isolates from Bihar. RNA2 sequence of PPSMV-1 isolate from Pune (KX363907) is also available.

**Table S2**. Primers used for RT-PCR amplification of different RNA segments of PPSMV-1 and PPSMV-2 isolates and their nucleotide coordinates.

| Primer Name | Primer Sequence (5’-3’) | RNA Segment | Nucleotide  position |
| --- | --- | --- | --- |
| PPSMV2-RNA1F  (Elbeaino et al*.*, 2015) | ATCAATACTCCATAGTGCACCTN | RNA-1 | 261-283 |
| PPSMV2-RNA1R  (Elbeaino et al*.*, 2015) | ACACCAACAGAAATATTCTTGGTGN | RNA-1 | 593-569 |
| PPSMV1&2-F | CCTCCTAAGAGATCANTCAGN | RNA-1 | 105-125 |
| PPSMV1&2-R | ATGAATCCTGTTCATTTTCN | RNA-1 | 845-826 |
| PPSMV1.NP-F | ACCGCTCATACATACATCTAATCAGC | RNA-3 | 70-96 |
| PPSMV1.NP-R | AAGAAGCACAACTTAAAGGCAAACTN | RNA-3 | 1068-1043 |
| PPSMV2.NP-F | ACCTGGTGTTTATTCCTCTCAAAGN | RNA-3 | 57-81 |
| PPSMV2.NP-R | GTGACATTAAATCACTAAGGCAAGN | RNA-3 | 1060-1036 |
| PPSMV.mp1&2-F | ATGATGCCTAGCACCTCCTATGN | RNA-4 | 1-23 |
| PPSMV.mp1&2-R | TATCTATATATATATAGATATGAN | RNA-4 | 1041-1018 |
| SMD6-F | AGTAGTGAGCTCCCTATAACAAGN | RNA-6 | 1-24 |
| SMD6-R | AGTAGTGTTCTCCCTATAAACAAAAN | RNA-6 | 1194-1169 |

Note: The primer pair PPSMV2-RNA1F and PPSMV2-RNA1R is specific to RNA1 of PPSMV-2 and are derived from Elbeaino et al. (2015).

**Table S3**. Percent nucleotide sequence identities of nucleocapsid protein (NP) (lower diagonal) of PPSMV-1 and PPSMV-2 (Grey shade) isolates and their corresponding amino acid sequence identities (upper diagonal).

|  | **1** | **2** | **3** | **4** | **5** | **6** | **7** | **8** | **9** | **10** | **11** | **12** | **13** | **14** | **15** | **16** | **17** | **18** | **19** | **FMV** |
| --- | --- | --- | --- | --- | --- | --- | --- | --- | --- | --- | --- | --- | --- | --- | --- | --- | --- | --- | --- | --- |
| **1** |  | **100.0** | 97.4 | 99.0 | 95.1 | 95.7 | 98.0 | 100.0 | 99.0 | 99.3 | 39.1 | 38.8 | 40.1 | 40.9 | 39.8 | 40.1 | 39.4 | 40.1 | 41.1 | 39.9 |
| **2** | **99.1** |  | 97.4 | 99.0 | 95.1 | 95.7 | 98.0 | 100.0 | 99.0 | 99.3 | 39.1 | 38.8 | 40.1 | 40.9 | 39.8 | 40.1 | 39.4 | 40.1 | 41.1 | 39.9 |
| **3** | 98.0 | 97.3 |  | 96.4 | 92.5 | 93.5 | 95.4 | 97.4 | 96.4 | 96.7 | 38.5 | 38.2 | 39.4 | 40.2 | 39.1 | 39.4 | 38.8 | 39.4 | 40.5 | 39.3 |
| **4** | 94.7 | 94.5 | 93.5 |  | 94.8 | 95.4 | 97.7 | 99.0 | **100.0** | 99.0 | 39.4 | 39.1 | 40.4 | 41.2 | 40.1 | 40.4 | 39.8 | 40.4 | 41.4 | 40.2 |
| **5** | 88.2 | 88.1 | 87.0 | 91.7 |  | **91.5** | 94.4 | 95.1 | 94.8 | 95.7 | 39.5 | 39.2 | 40.5 | 41.3 | 40.5 | 40.5 | 39.8 | 40.5 | 41.5 | 40.6 |
| **6** | 96.7 | 96.6 | 96.4 | 92.8 | **86.5** |  | 94.4 | 95.7 | 95.4 | 95.7 | 38.2 | 37.9 | 39.1 | 40.2 | 38.8 | 39.1 | 38.5 | 39.1 | 40.1 | 38.6 |
| **7** | 94.0 | 94.0 | 93.0 | 97.8 | 91.4 | 92.3 |  | 98.0 | 97.7 | 98.7 | 39.4 | 39.1 | 40.4 | 41.5 | 40.1 | 40.4 | 39.8 | 40.4 | 41.4 | 40.2 |
| **8** | 98.7 | 98.4 | 96.7 | 94.4 | 88.2 | 96.0 | 93.7 |  | 99.0 | 99.3 | 39.1 | 38.8 | 40.1 | 40.9 | 39.8 | 40.1 | 39.4 | 40.1 | 41.1 | 39.9 |
| **9** | 98.0 | 97.8 | 96.8 | 95.7 | 88.0 | 96.3 | 94.3 | 97.7 |  | 99.0 | 39.4 | 39.1 | 40.4 | 41.2 | 40.1 | 40.4 | 39.8 | 40.4 | 41.4 | 40.2 |
| **10** | 94.6 | 94.6 | 93.4 | 98.4 | 91.6 | 92.7 | 99.1 | 94.3 | 94.9 |  | 39.4 | 39.1 | 40.4 | 41.2 | 40.1 | 40.4 | 39.8 | 40.4 | 41.4 | 40.2 |
| **11** | 51.0 | 51.0 | 50.4 | 52.7 | 51.3 | 49.8 | 52.9 | 50.7 | 50.5 | 53.2 |  | **99.6** | 98.0 | 92.0 | 95.8 | 95.5 | 95.8 | 96.1 | 93.9 | 79.2 |
| **12** | 50.7 | 50.7 | 50.3 | 52.6 | 51.2 | 49.7 | 52.8 | 50.4 | 50.4 | 52.9 | 98.8 |  | 97.7 | 91.7 | 95.5 | 95.2 | 95.5 | 95.8 | 93.6 | 78.9 |
| **13** | 51.0 | 51.0 | 50.4 | 52.7 | 51.3 | 49.8 | 52.9 | 50.7 | 50.5 | 53.2 | **99.8** | 98.7 |  | 93.3 | 97.1 | 96.8 | 96.8 | 97.4 | 95.2 | 80.5 |
| **14** | 49.4 | 49.5 | 48.8 | 51.3 | 50.2 | 48.4 | 51.1 | 49.2 | 49.1 | 51.2 | 93.0 | 92.0 | 92.9 |  | 91.4 | 91.0 | **90.7** | 91.7 | 94.1 | 76.3 |
| **15** | 50.0 | 50.1 | 50.0 | 52.5 | 51.4 | 49.4 | 52.4 | 50.2 | 50.2 | 52.5 | 92.5 | 92.2 | 92.4 | 87.1 |  | 96.8 | 96.8 | 96.8 | 94.9 | 79.6 |
| **16** | 51.0 | 51.0 | 50.6 | 53.0 | 51.2 | 50.1 | 53.3 | 50.6 | 50.8 | 53.4 | 92.8 | 92.0 | 92.7 | 86.8 | 93.6 |  | 98.7 | 96.1 | 94.2 | 78.9 |
| **17** | 50.8 | 50.8 | 50.4 | 52.8 | 51.0 | 49.9 | 53.1 | 50.4 | 50.6 | 53.2 | 92.8 | 92.0 | 92.7 | **86.7** | 93.6 | 99.3 |  | 96.1 | 93.9 | 78.9 |
| **18** | 50.7 | 50.8 | 50.3 | 52.4 | 51.9 | 50.0 | 52.8 | 50.4 | 50.3 | 53.1 | 92.7 | 91.9 | 92.6 | 86.9 | 92.9 | 92.3 | 92.3 |  | 96.8 | 80.2 |
| **19** | 51.3 | 51.4 | 50.9 | 53.2 | 52.4 | 50.6 | 53.1 | 51.0 | 51.0 | 53.4 | 90.4 | 89.6 | 90.3 | 89.7 | 90.2 | 90.1 | 90.0 | 95.1 |  | 79.2 |
| **FMV** | 47.8 | 48.0 | 47.6 | 50.3 | 49.7 | 47.4 | 49.8 | 47.7 | 48.1 | 50.0 | 69.5 | 68.8 | 69.4 | 70.2 | 70.1 | 69.5 | 69.4 | 69.9 | 71.3 |  |

**Note:** Highest and lowest identities among the isolates of PPSMV-1 and PPSMV-2 are shown in bold fonts. The PPSMV-1 and PPSMV-2 isolates used in this study are: (**1**). PPSMV1-Bengaluru.3, (**2**). PPSMV1-Bengaluru.6, (**3**). PPSMV1-Bengaluru.7, (**4**). PPSMV1-Bidar, (**5**). PPSMV1-Bihar, (**6**). PPSMV1-Gorakhpur, (**7**). PPSMV1-Kalaburagi, (**8**). PPSMV1-Mahagaon, (**9**). PPSMV1-Pune, (**10**). PPSMV1-Patancheru, (**11**). PPSMV2-Bengaluru.2, (**12**). PPSMV2-Bengaluru.5, (**13**). PPSMV2-Bengaluru.8, (**14**). PPSMV2-Bengaluru.10, (**15**). PPSMV2-Bihar, (**16**). PPSMV2-Coimbatore, (**17**). PPSMV2-Delhi.2, (**18**). PPSMV2-Raichur, (**19**). PPSMV2-Patancheru, FMV (NCBI Accession No. HQ703345). All the PPSMV-1 (#1-10) and PPSMV-2 (#11-19) isolates except for Patancheru in Telangana (Elbeaino et al., 2014, 2015) are from this study.

**Table S4**. Percent nucleotide sequence identities of the putative movement protein (MP) (lower diagonal) of PPSMV-1 and PPSMV-2 (Grey shade) isolates and their corresponding amino acid sequence identities (upper diagonal).

|  | **1** | **2** | **3** | **4** | **5** | **6** | **7** | **8** | **9** | **10** | **11** | **12** | **13** | **14** | **15** | **16** | **17** | **18** | **19** | **20** | **21** | **22** | **23** | **24** | **25** | **FMV** |
| --- | --- | --- | --- | --- | --- | --- | --- | --- | --- | --- | --- | --- | --- | --- | --- | --- | --- | --- | --- | --- | --- | --- | --- | --- | --- | --- |
| **1** |  | 98.6 | 98.0 | 99.4 | 97.5 | 98.8 | **100.0** | 98.8 | 98.3 | 99.1 | 96.6 | 99.1 | 98.3 | 95.0 | 96.1 | 97.2 | 98.6 | 98.8 | 40.3 | 40.9 | 40.1 | 40.1 | 40.6 | 40.1 | 40.6 | 38.4 |
| **2** | 97.7 |  | 98.8 | 99.1 | 98.3 | 99.7 | 98.6 | 99.1 | 98.6 | 99.4 | 97.5 | 99.4 | 98.6 | 95.8 | 96.9 | 98.0 | 99.4 | 99.7 | 40.6 | 41.2 | 40.3 | 40.3 | 40.9 | 40.3 | 40.9 | 38.7 |
| **3** | 94.6 | 95.8 |  | 98.6 | 99.4 | 98.6 | 98.0 | 98.6 | 98.0 | 98.8 | 98.6 | 98.8 | 98.0 | 96.1 | 96.6 | 97.5 | 98.8 | 99.1 | 40.3 | 40.9 | 40.6 | 40.1 | 40.6 | 40.1 | 40.6 | 38.7 |
| **4** | 99.5 | 97.9 | 94.9 |  | 98.0 | 99.4 | 99.4 | 99.4 | 98.8 | 99.7 | 97.2 | 99.7 | 98.8 | 95.5 | 96.6 | 97.7 | 99.1 | 99.4 | 40.3 | 40.9 | 40.1 | 40.1 | 40.6 | 40.1 | 40.6 | 38.4 |
| **5** | 94.4 | 95.5 | 99.6 | 94.7 |  | 98.0 | 97.5 | 98.0 | 97.5 | 98.3 | 98.0 | 98.3 | 97.5 | 95.5 | 96.1 | 96.9 | 98.3 | 98.6 | 40.3 | 40.9 | 40.6 | 40.1 | 40.6 | 40.1 | 40.6 | 38.7 |
| **6** | 98.2 | 99.4 | 95.6 | 98.5 | 95.4 |  | 98.8 | 99.4 | 98.8 | 99.7 | 97.2 | 99.7 | 98.8 | 95.5 | 96.6 | 97.7 | 99.1 | 99.4 | 40.6 | 41.2 | 40.3 | 40.3 | 40.9 | 40.3 | 40.9 | 38.7 |
| **7** | **100.0** | 97.7 | 94.6 | 99.5 | 94.4 | 98.2 |  | 98.8 | 98.3 | 99.1 | 96.6 | 99.1 | 98.3 | 95.0 | 96.1 | 97.2 | 98.6 | 98.8 | 40.3 | 40.9 | 40.1 | 40.1 | 40.6 | 40.1 | 40.6 | 38.4 |
| **8** | 99.5 | 97.9 | 94.9 | 99.6 | 94.7 | 98.5 | 99.5 |  | 98.8 | 99.7 | 97.2 | 99.7 | 98.8 | 95.5 | 96.6 | 97.7 | 99.1 | 99.4 | 40.6 | 41.2 | 40.3 | 40.3 | 40.9 | 40.3 | 40.9 | 38.7 |
| **9** | 99.0 | 97.7 | 94.7 | 99.1 | 94.5 | 98.2 | 99.0 | 99.3 |  | 99.1 | 96.6 | 99.1 | **100.0** | 95.0 | 96.1 | 97.2 | 98.6 | 98.8 | 39.8 | 40.6 | 39.8 | 39.8 | 40.3 | 39.8 | 40.3 | 38.1 |
| **10** | 99.6 | 98.0 | 95.0 | 99.7 | 94.8 | 98.6 | 99.6 | 99.9 | 99.4 |  | 97.5 | **100.0** | 99.1 | 95.8 | 96.9 | 98.0 | 99.4 | 99.7 | 40.6 | 41.2 | 40.3 | 40.3 | 40.9 | 40.3 | 40.9 | 38.7 |
| **11** | 91.5 | 92.0 | 94.2 | 91.8 | 94.2 | 92.0 | 91.5 | 91.8 | 91.6 | 91.9 |  | 97.5 | 96.6 | **94.7** | 95.2 | 96.1 | 97.5 | 97.7 | 40.3 | 40.9 | 40.6 | 40.1 | 40.6 | 40.1 | 40.6 | 38.7 |
| **12** | 99.6 | 98.0 | 95.0 | 99.7 | 94.8 | 98.6 | 99.6 | 99.9 | 99.4 | 100.0 | 91.9 |  | 99.1 | 95.8 | 96.9 | 98.0 | 99.4 | 99.7 | 40.6 | 41.2 | 40.3 | 40.3 | 40.9 | 40.3 | 40.9 | 38.7 |
| **13** | 99.1 | 97.6 | 94.6 | 99.2 | 94.4 | 98.1 | 99.1 | 99.4 | 99.9 | 99.5 | 91.5 | 99.5 |  | 95.0 | 96.1 | 97.2 | 98.6 | 98.8 | 39.8 | 40.6 | 39.8 | 39.8 | 40.3 | 39.8 | 40.3 | 38.1 |
| **14** | 97.0 | 96.5 | 93.8 | 97.3 | 93.6 | 96.5 | 97.0 | 97.3 | 96.8 | 97.4 | **90.8** | 97.4 | 96.9 |  | 98.8 | 95.0 | 96.3 | 96.1 | 41.4 | 40.6 | 40.3 | 40.3 | 40.3 | 39.8 | 40.3 | 39.0 |
| **15** | 97.7 | 96.8 | 94.1 | 97.9 | 93.9 | 97.2 | 97.7 | 97.9 | 97.5 | 98.0 | 91.3 | 98.0 | 97.6 | 98.6 |  | 95.5 | 97.5 | 97.2 | 41.7 | 40.9 | 40.1 | 40.6 | 40.6 | 40.1 | 40.6 | 39.0 |
| **16** | 97.9 | 97.3 | 94.4 | 98.0 | 94.2 | 97.3 | 97.9 | 98.2 | 97.7 | 98.3 | 91.4 | 98.3 | 97.8 | 97.4 | 97.1 |  | 98.0 | 98.3 | 40.6 | 41.2 | 40.3 | 40.3 | 40.9 | 40.3 | 40.9 | 38.7 |
| **17** | 96.3 | 97.6 | 96.2 | 96.5 | 95.9 | 97.2 | 96.3 | 96.5 | 96.3 | 96.6 | 91.9 | 96.6 | 96.2 | 95.3 | 95.5 | 96.1 |  | 99.7 | 40.9 | 40.9 | 40.1 | 40.1 | 40.6 | 40.1 | 40.6 | 38.7 |
| **18** | 98.8 | 97.9 | 95.1 | 98.8 | 94.9 | 98.1 | 98.8 | 99.0 | 98.6 | 99.1 | 92.4 | 99.1 | 98.7 | 97.6 | 98.1 | 98.4 | 96.9 |  | 40.6 | 41.2 | 40.3 | 40.3 | 40.9 | 40.3 | 40.9 | 38.7 |
| **19** | 51.9 | 52.2 | 52.1 | 52.1 | 52.1 | 52.3 | 51.9 | 52.1 | 51.8 | 52.2 | 52.1 | 52.2 | 51.8 | 52.5 | 52.0 | 52.7 | 52.5 | 52.3 |  | 97.2 | 96.6 | **96.3** | 97.5 | 96.6 | 97.5 | 73.6 |
| **20** | 51.3 | 51.6 | 51.5 | 51.5 | 51.5 | 51.7 | 51.3 | 51.5 | 51.3 | 51.6 | 51.3 | 51.6 | 51.3 | 51.5 | 51.0 | 52.1 | 51.9 | 51.7 | 96.5 |  | 98.8 | 97.5 | 99.1 | 98.8 | **99.7** | 75.0 |
| **21** | 51.1 | 51.0 | 51.0 | 51.3 | 51.0 | 51.1 | 51.1 | 51.3 | 51.1 | 51.4 | 51.0 | 51.4 | 51.1 | 51.1 | 50.5 | 51.6 | 51.4 | 51.1 | **94.1** | 95.1 |  | 96.9 | 98.6 | 98.3 | 99.1 | 75.0 |
| **22** | 51.4 | 51.7 | 51.2 | 51.6 | 51.2 | 51.8 | 51.4 | 51.6 | 51.4 | 51.7 | 50.8 | 51.7 | 51.4 | 51.8 | 51.3 | 52.2 | 51.8 | 51.8 | 96.2 | 96.3 | 94.4 |  | 97.7 | 96.9 | 97.7 | 73.6 |
| **23** | 51.5 | 51.8 | 51.7 | 51.7 | 51.7 | 51.8 | 51.5 | 51.7 | 51.5 | 51.8 | 51.7 | 51.8 | 51.5 | 51.7 | 51.2 | 52.3 | 52.1 | 51.8 | 98.0 | 97.7 | 95.1 | 97.6 |  | 98.6 | 99.4 | 74.7 |
| **24** | 51.3 | 51.6 | 51.4 | 51.5 | 51.4 | 51.7 | 51.3 | 51.5 | 51.3 | 51.6 | 51.2 | 51.6 | 51.3 | 51.5 | 51.0 | 52.1 | 51.9 | 51.7 | 96.5 | **99.0** | 94.8 | 96.2 | 97.6 |  | 99.1 | 75.0 |
| **25** | 51.5 | 51.6 | 51.5 | 51.7 | 51.5 | 51.7 | 51.5 | 51.7 | 51.5 | 51.8 | 51.4 | 51.8 | 51.5 | 51.5 | 51.0 | 52.1 | 51.9 | 51.7 | 97.0 | 98.2 | 95.5 | 97.3 | 98.2 | 98.1 |  | 75.0 |
| **FMV** | 34.4 | 34.4 | 34.6 | 34.6 | 34.6 | 34.4 | 34.4 | 34.5 | 34.5 | 34.6 | 34.7 | 34.6 | 34.4 | 34.6 | 34.2 | 34.9 | 34.7 | 34.6 | 54.1 | 54.7 | 55.2 | 53.7 | 54.6 | 54.7 | 54.7 |  |

**Note:** Highest and lowest identities among the isolates of PPSMV-1 and PPSMV-2 are shown in bold fonts. All the PPSMV-1 and PPSMV-2 isolates except for Patancheru in Telangana (Elbeaino et al., 2014, 2015) are from this study. The PPSMV-1 and PPSMV-2 isolates used in this study are: (**1**). PPSMV1-Bengaluru.1, (**2**). PPSMV1-Bengaluru.2, (**3**). PPSMV1-Bengaluru.3, (**4**). PPSMV1-Bengaluru.4, (**5**). PPSMV1-Bengaluru.6, (**6**). PPSMV1-Bengaluru.7, (**7**). PPSMV1-Bengaluru.8, (**8**). PPSMV1-Bengaluru.9, (**9**). PPSMV1-Bengaluru.10, (**10**). PPSMV1-Bidar, (**11**). PPSMV1-Bihar, (**12**). PPSMV1-Coimbatore.2, (**13**). PPSMV1-Delhi.1, (**14**). PPSMV1-Gorakhpur, (**15**). PPSMV1-Kalaburagi, (**16**). PPSMV1-Mahagaon, (**17**). PPSMV1-Pune, (**18**). PPSMV1-Patancheru, (**19**). PPSMV2-Bengaluru.5, (**20**). PPSMV2-Bengaluru.8, (**21**). PPSMV2-Bihar, (**22**). PPSMV2-Coimbatore, (**23**). PPSMV2-Mahagaon, (**24**). PPSMV2-Raichur, (**25**). PPSMV2-Patancheru, and FMV (NCBI Accession No. HQ703346).

**Supplementary Figures:**

**Figure S1**. Symptoms of sterility mosaic disease (SMD)-affected pigeonpea from India used in this study. For each SMD sample, the PPSMV species involved is indicated.


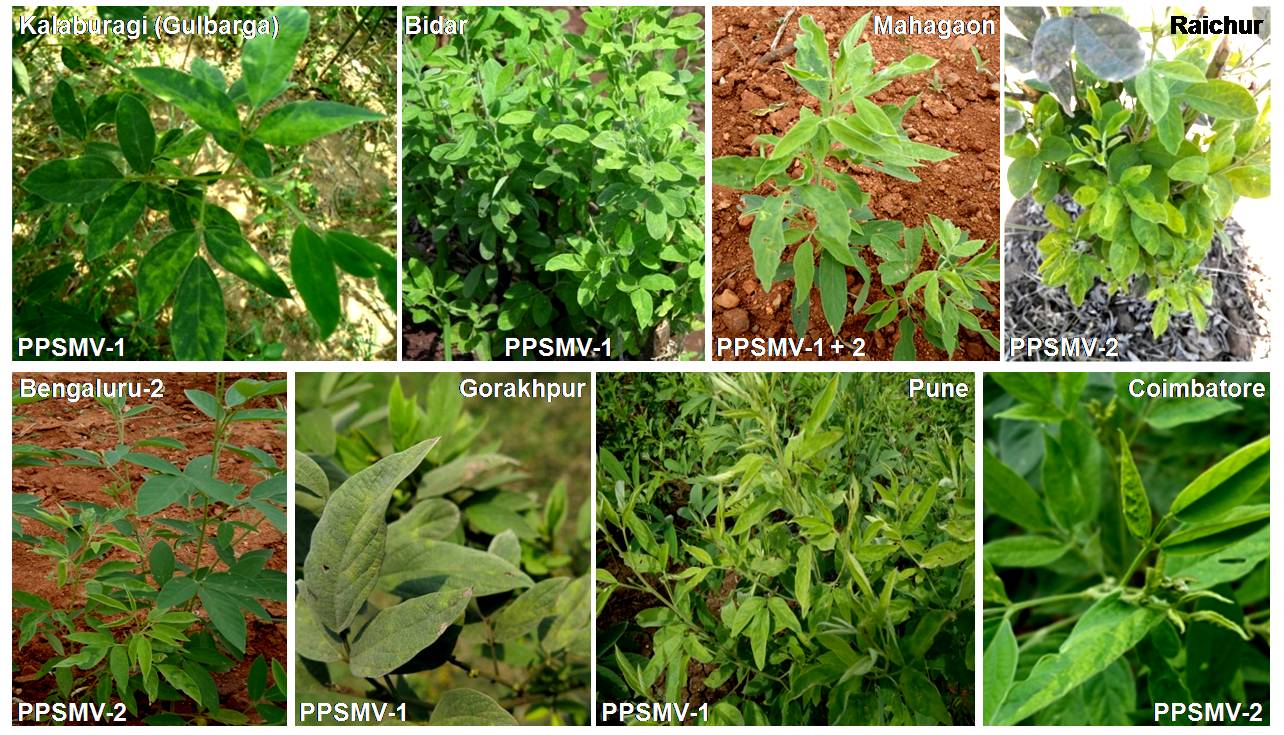


**Figure S2.** Map of India showing the ten locations of pigeonpea samples collected and distribution of PPSMV-1 and PPSMV-2. Additionally, samples with mixed infections or reassortment are indicated. The data for Patancheru in Telangana state comes from Elbeaino et al. (Elbeaino et al., 2014, 2015).


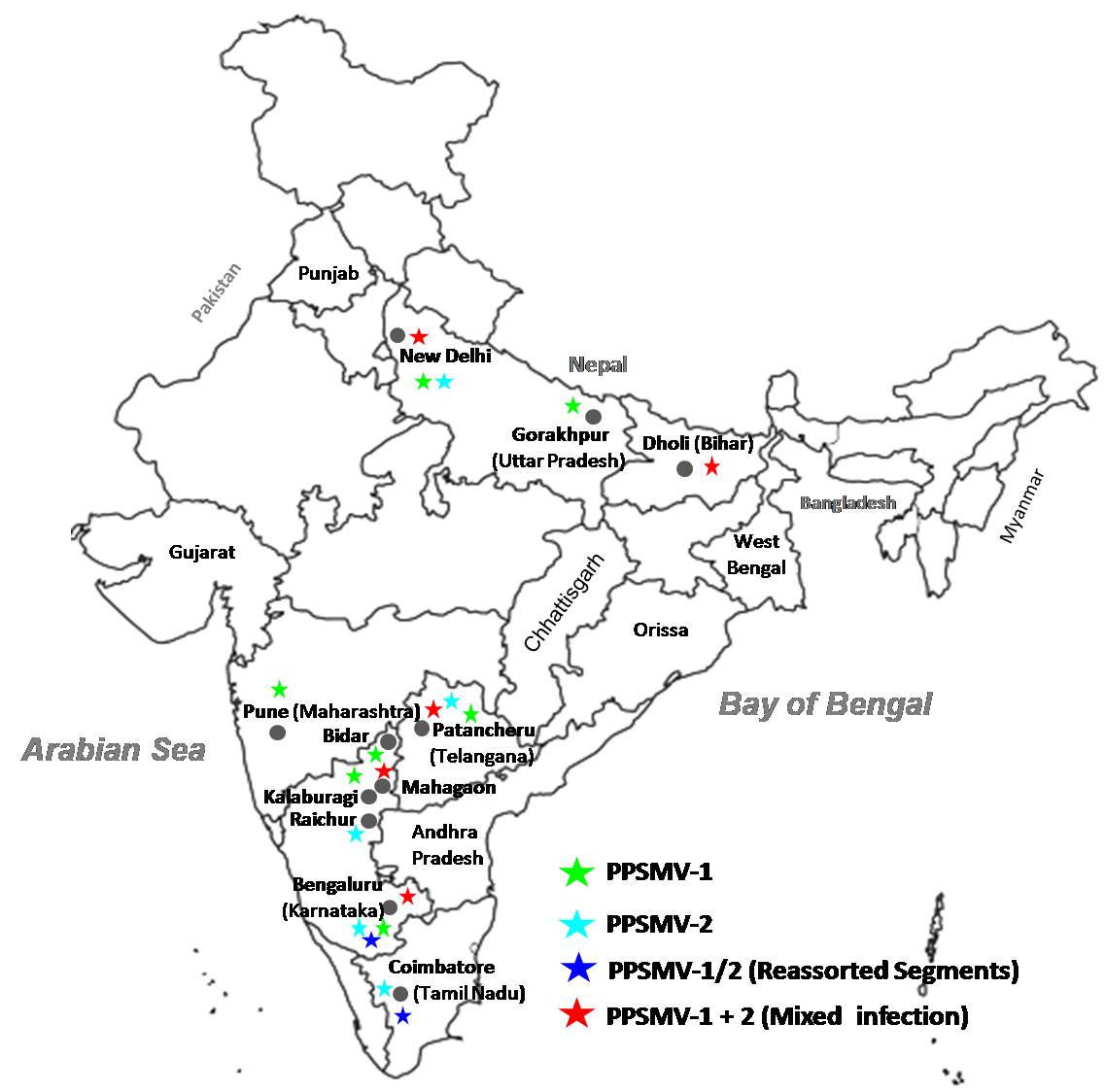


**Figure S3.** Phylogenetic analysis of nucleotide sequences of RNA5 and RNA6 segments of PPSMV-1 and 2 isolates, along with corresponding sequences of selected emaraviruses such as *Fig mosaic emaravirus* (FMV) and *Raspberry leaf blotch emaravirus* (RLBV)*.* The accession number for each sequence is given in parentheses. Sequence alignments were done using ‘‘MUSCLE’’ and the phylogenetic trees were drawn by the maximum-likelihood, applying the JTT matrix and pairwise gap deletion options implemented in MEGA6, using 1000 bootstrap replicates. Bootstrap values greater than 70% are given at each node. The scale bar represents 0.05 and 0.1 substitutions per nucleotide position for RNA5 and RNA6 respectively.

**
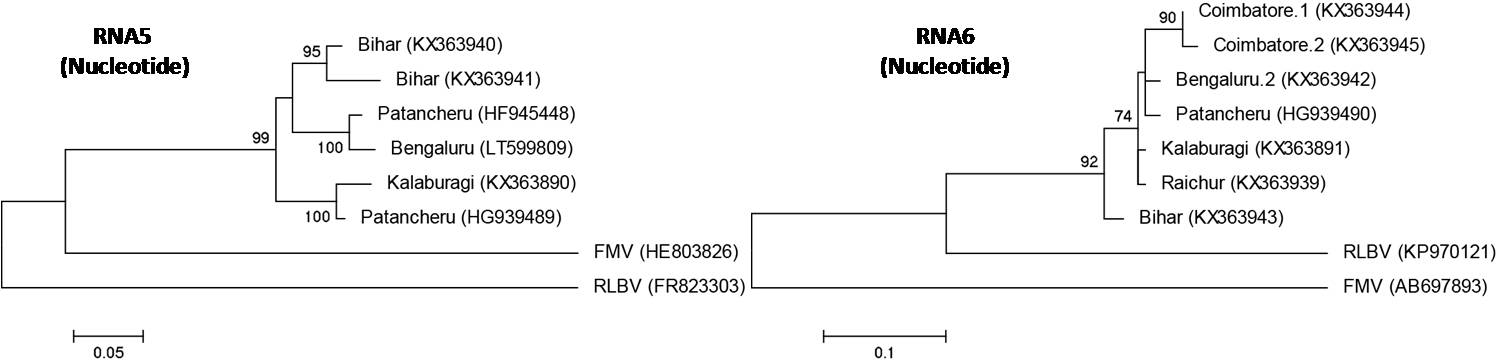
**
